# Gut microbiome of the sole surviving member of reptile order Rhynchocephalia reveals biogeographic variation, influence of host body condition and a substantial core microbiota in tuatara across New Zealand

**DOI:** 10.1002/ece3.11073

**Published:** 2024-02-22

**Authors:** Carmen Hoffbeck, Danielle M. R. L. Middleton, Sarah K. Lamar, Susan N. Keall, Nicola J. Nelson, Michael W. Taylor

**Affiliations:** ^1^ School of Biological Sciences University of Auckland Auckland New Zealand; ^2^ Manaaki Whenua – Landcare Research Lincoln New Zealand; ^3^ School of Biological Sciences Victoria University of Wellington Wellington New Zealand

**Keywords:** microbial ecology, microbiome, reptile, translocation

## Abstract

Tuatara are the sole extant species in the reptile order Rhynchocephalia. They are ecologically and evolutionarily unique, having been isolated geographically for ~84 million years and evolutionarily from their closest living relatives for ~250 million years. Here we report the tuatara gut bacterial community for the first time. We sampled the gut microbiota of translocated tuatara at five sanctuaries spanning a latitudinal range of ~1000 km within Aotearoa New Zealand, as well as individuals from the source population on Takapourewa (Stephens Island). This represents a first look at the bacterial community of the order Rhynchocephalia and provides the opportunity to address several key hypotheses, namely that the tuatara gut microbiota: (1) differs from those of other reptile orders; (2) varies among geographic locations but is more similar at sites with more similar temperatures and (3) is shaped by tuatara body condition, parasitism and ambient temperature. We found significant drivers of the microbiota in sampling site, tuatara body condition, parasitism and ambient temperature, suggesting the importance of these factors when considering tuatara conservation. We also derived a ‘core’ community of shared bacteria across tuatara at many sites, despite their geographic range and isolation. Remarkably, >70% of amplicon sequence variants could not be assigned to known genera, suggesting a largely undescribed gut bacterial community for this ancient host species.

## Graphical abstract

Tuatara, an endemic reptile to New Zealand, are a unique species in both their evolution and ecology, but have declined due to habitat loss and invasive predators. We here examine their microbial community to better understand their ecology and support their conservation. We found 70% of the tuatara gut to be populated with unknown bacterial genera and found significant impacts of temperature, parasitism and tuatara body condition on the tuatara gut community.

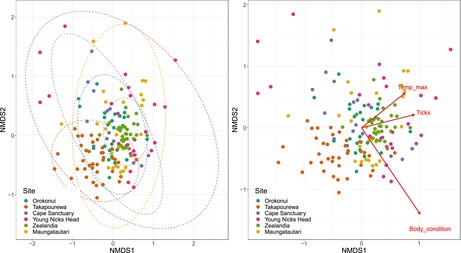

## 1 INTRODUCTION

The community of microorganisms living on and within other organisms, collectively known as the microbiota, is increasingly recognised as a major driver of host digestion, immunity and behaviour (Colston & Jackson, 2016; McFall‐Ngai et al., 2013; Song et al., 2020; Wang et al., 2017). While these diverse and important ecosystem members have been extensively studied for some host taxa, the microbiology of reptiles has rarely been considered, beyond a focus on their potential pathogens (Jacobson, 2007; Lamm et al., 1972; Warwick et al., 2001). However, increasing attention on the entire bacterial community of reptiles has led to identification of a diverse and varied community living in the reptile gut (Hoffbeck et al., 2023). A range of factors are now known to influence reptile gut microbiotas, including stressors such as high temperature, disease and pollution (Biagi et al., 2021; Indest et al., 2018; Khan et al., 2021; Madison et al., 2018; McNally et al., 2021; Moeller et al., 2020; Zhang et al., 2022). Geographic distribution also influences the reptile gut microbiota (Baldo et al., 2018; Price et al., 2017; Zhang et al., 2021), as do life stage and diet (Campos et al., 2019; Du et al., 2022; Holmes et al., 2019; Hong et al., 2021; Kohl et al., 2014; Peng et al., 2020; Tang, Wang, et al., 2019; Tang, Zhu, et al., 2019b; Youngblut et al., 2019) and, in at least some cases, host phylogeny (Hoffbeck et al., 2023; Song et al., 2020). However, research on bacterial communities from reptiles has so far only included members of the orders Squamata, Testudines and Crocodilia, and is yet to consider the fourth reptile order, Rhynchocephalia.

Unique among reptiles, the tuatara is the last surviving member of order Rhynchocephalia, having diverged from their closest living relatives 250 million years ago (Hugall et al., 2007). Endemic to New Zealand, they are evolutionarily distinct, as a monotypic species which has not speciated and has morphologically evolved very little over the ~84 million years since the continent of Zealandia (containing New Zealand) split from Gondwana (Gemmell et al., 2020; Mortimer et al., 2019). One might surmise that during this time tuatara have developed a unique microbiota, and indeed, this is suggested by the number of novel viruses present in the tuatara gut (Waller et al., 2022). The evolutionary status of tuatara represents an opportunity to examine phylosymbiosis—‘microbial community relationships that recapitulate the phylogeny of their host’ (Lim & Bordenstein, 2020)—of the reptile community. As tuatara are the closest relatives of the squamates (lizards, snakes, amphisbaenians), their microbiota may be expected to resemble most closely that of lizards and other squamates. Alternatively, their status as an ancient and relatively unchanged species may reflect a bacterial community more like the ancestral state for reptiles, perhaps harbouring new or unexpected bacterial species relative to other vertebrates.

Tuatara are ecologically as well as evolutionarily unique. Despite the near‐ubiquity of the bacterial genus *Salmonella* among reptiles (Middleton et al., 2014), tuatara do not appear to harbour *Salmonella*, even when sharing burrows with seabirds that do (Middleton et al., 2015). This apparent resistance is thought to be due to an innate gut mucosal immune response (Middleton et al., 2015), perhaps indicating a unique microbial community for the tuatara. Once widespread throughout the mainland of New Zealand, tuatara are now confined to offshore islands and protected mainland sanctuaries due to intense predation pressure from introduced mammals and habitat loss. Aside from translocations to these sanctuaries around New Zealand, and their existence in zoos, the extant tuatara population is now entirely located on 32 offshore islands, primarily Takapourewa (Stephens Island), in New Zealand's Cook Strait. The pockets of translocated tuatara represent small, geographically isolated populations. Isolation is known to introduce population‐level differences in the microbiota of other reptile species (Alemany et al., 2022), while diet and environment are also acknowledged drivers of gut microbiota composition (Grond et al., 2019; Jin et al., 2021; Waite & Taylor, 2014; Youngblut et al., 2019). This may introduce variation in the gut microbiota of tuatara from different sites around New Zealand.

Tuatara are cold‐adapted reptiles, with a maximum thermal tolerance near 30°C (Heatwole, 1982). Temperature is a known driver of gut microbiota composition in other reptile species (Moeller et al., 2020; Zhang et al., 2022) and may be an even stronger driver for a species adapted to colder climates. This carries particular relevance for tuatara conservation, with 30°C now regularly reached in parts of New Zealand inhabited by tuatara. Although the risk of tuatara overheating is alleviated somewhat by hunting primarily at night and by dwelling and caching their eggs in underground burrows, it is nonetheless important to understand how temperature may impact the tuatara. With expected mid‐century temperatures of +1.1°C due to climate change (IPCC, 2021), temperature will only increase in its importance to tuatara conservation.

Tuatara occupy a fascinating position in science for their ecological and evolutionary uniqueness, as well as holding great cultural importance for Māori, the indigenous people of New Zealand, and for conservation. In this study, we sampled the gut microbiota of translocated tuatara at five sanctuaries spanning a latitudinal range of ~1000 km within New Zealand, as well as sampling individuals from the source population on Takapourewa. This represents the first look at the bacterial community of the order Rhynchocephalia and affords us the opportunity to address several key hypotheses, namely that the tuatara gut microbiota: (1) differs from those of other reptile orders but most closely resembles those of the squamates; (2) varies among geographic locations but is more similar at sites containing similar habitats and (3) is shaped by tuatara body condition, parasitism and ambient temperature. Finally, this research should help inform the conservation management of tuatara in sanctuaries and captivity, as parasitism and temperature are intrinsically site dependent. As the sole representative of the fourth reptilian order and an at‐risk species, tuatara remain an important link to the ancient world and the microbiota that their extinct relatives may have carried.

## 2 MATERIALS AND METHODS

### 2.1 Sample collection

**FIGURE 1:**
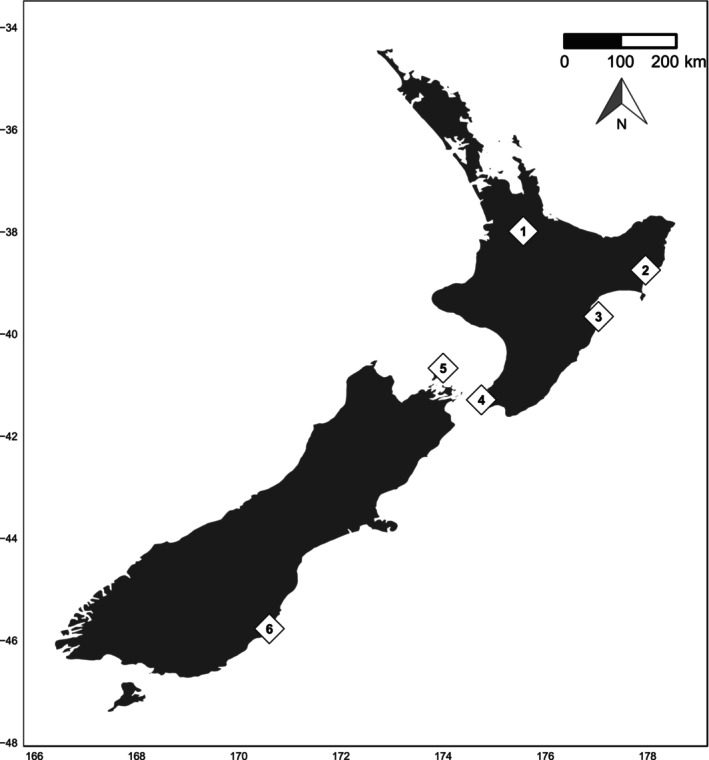
Sampling sites for tuatara in February–March 2022 and January–February 2023: (1) Sanctuary Mountain Maungatautari, (2) Young Nick's Head, (3) Cape Sanctuary, (4) Zealandia Ecosanctuary, (5) Takapourewa (Stephens Island), (6) Orokonui Ecosanctuary.

Tuatara were sampled during the austral summer: samples from Takapourewa were collected in February–March 2022, with all other samples collected from Zealandia Ecosanctuary, Sanctuary Mountain Maungatautari, Young Nick's Head, Cape Sanctuary and Orokonui Ecosanctuary in January–February 2023 (Figure 1). To obtain a proxy for the gut microbial community, all tuatara were captured by hand and sampled via cloacal swabbing. A small, sterile cotton‐tipped swab was inserted 20 mm into the cloaca and rotated gently on the cloacal wall before being removed and placed into RNAlater for storage at −20°C (~4°C for Takapourewa tuatara). Two swabs were collected from each animal, except for those on Takapourewa from which a single swab was collected. Though cloacal swabs provide only a proxy for the gut microbial community and may lead to collection of lower microbial biomass, this method was deemed most appropriate for our study species. Animal ethics approval was granted by the Victoria University of Wellington (Permission #30011), and research approval was given by the Department of Conservation (Authorisation 50568‐FAU).

Each animal was identified, if possible, by PIT tag or historic toe clips, and was demarcated by xylene‐free marker pen if no other identification was present to avoid repeated sampling of the same individual. Once identified, the sex and life stage of each animal were estimated using secondary sex characteristics and the presence and count of cutaneous ticks and mites were determined. For tick abundance, the tick number was rounded to the nearest 10. Each animal was then weighed by spring balance and measured for snout‐vent length (SVL), vent‐tail length (VT) and tail regrowth. Snout‐vent length and VT were summed to obtain total length, and body condition was calculated as $\frac{\log\left(\text{weight}\right)}{\log\left(\mathrm{SVL}\right)}$ (Lamar et al., 2022). The average daily maximum and minimum temperatures for each site during the sampling period were obtained using the nearest National Institute of Water and Atmospheric Research (NIWA) stations through the NIWA CliFlo database.

### 2.2 DNA extraction, 16S rRNA gene amplification and sequencing

All swabs were removed from RNAlater and DNA was extracted using a QIAGEN QIAamp Fast DNA Stool Kit, as recommended by the manufacturer for bacterial DNA collection. Though this kit is designed for faecal input, its efficacy for tuatara DNA extraction had been previously determined for a single swab input before sampling. Swabs from each site were extracted alongside an extraction blank containing no input material. Extracted DNA was stored at −20°C. The V3‐V4 region of the bacterial 16S rRNA gene was PCR‐amplified by using the 341F‐806R primer pair and the KAPA 3G kit, with the following thermal cycling conditions: initial denaturation at 95°C for 3 min, then 35 cycles of denaturation at 95°C for 20 s, annealing at 57°C for 15 s and extension at 72°C for 30 s, followed by a final extension at 72°C for 1 min (West et al., 2022). The presence of a correct‐sized amplicon was confirmed using 1% agarose gel electrophoresis, and DNA quantity was measured using an EnSpire Multimode Plate Reader. Following amplification, 10 μL of PCR product from each sample was purified using the Zymo ZR‐96 DNA Clean‐up Kit and sequenced on Illumina MiSeq (2 × 300 bp chemistry) by Auckland Genomics Ltd, together with one PCR‐amplified extraction blank from each sampling site, totalling 6 blanks.

### 2.3 Bioinformatics analysis

Sequence reads were processed into amplicon sequence variants (ASVs) using a DADA2‐based (Callahan et al., 2016) bioinformatics pipeline on the New Zealand eScience Infrastructure (NeSI) computing cluster. Illumina adaptors and primers were removed from each sample using Trimmomatic with standard settings (Bolger et al., 2014), with sequences then trimmed and filtered for quality and merged using DADA2 in R (Callahan et al., 2016). Taxonomy was assigned as far as genus level for the remaining samples using the SILVA 138 database (Quast et al., 2013), and verified with an additional taxonomic assignment step using DIAMOND BLAST alignment (Buchfink et al., 2021). ASVs which accounted for less than 0.001% of sequence reads across the entire data set were removed, as were 11 samples for which no reads remained after this filtering, merging and pruning process. Contaminant ASVs were removed prior to downstream analyses using the decontam package in R (Davis et al., 2018; ver 1.18.0) by identifying bacterial DNA present in extraction blanks with default threshold 0.1, which resulted in the identification of 125 contaminant ASVs. To normalise and compare across samples with different sampling depths, each sample was standardised to 1400 sequence reads using the SRS function in the SRS package (Heidrich et al., 2021; ver 0.2.3), which resulted in the removal of two further samples with low sequencing depth. Ultimately, 161 samples remained and were used for statistical analysis.

Bacterial community alpha‐diversity was calculated with the plot_richness function in phyloseq (McMurdie & Holmes, 2013; ver 1.42.0) using the observed diversity metric and confirmed using Shannon diversity, and significance was determined by the Wilcoxon test. Beta‐diversity was calculated using the ordinate function in phyloseq with Bray–Curtis dissimilarity and confirmed using weighted UniFrac (Lozupone et al., 2011), and visualised using non‐metric multidimensional scaling (nMDS). Significance was determined for discrete variables using the adonis2 PERMANOVA function in vegan (Oksanen et al., 2015; ver 2.6‐4) using Bray–Curtis dissimilarity with 9999 permutations. For continuous variables, the envfit function in vegan was used to determine significance and visualise effect sizes on the bacterial community. Taxonomy was visualised at the phylum and genus levels using ggplot2 (Wickham, 2016; ver 3.4.0). Jaccard similarity was calculated after merging samples by site and compared using the distance function in phyloseq. Members of the core microbiota were identified using the core_members function with an 80% prevalence threshold in microbiome (Lahti & Shetty, 2017; ver 1.20.0), both for all individuals and for individuals at each site. For comparison to other reptile species, data on reptile microbiotas were collected from publicly available databases and processed through a uniform bioinformatics pipeline, then assembled at the genus level for comparison (Hoffbeck et al., 2023).

## 3 RESULTS

### 3.1 Factors shaping the tuatara gut microbiota

**FIGURE 2:**
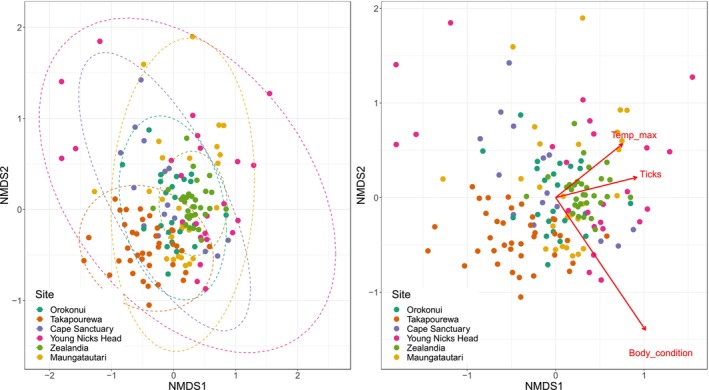
16S rRNA gene‐based nMDS Bray ordination showing effects on tuatara bacterial community composition of (a) sampling site, and (b) tuatara body condition, tick abundance and maximum temperature at the sampling site. Ellipses in (a) represent statistically similar communities at 95% confidence. Arrow length in (b) represents the strength of the *R* ^2^ value for that factor.

Although the tuatara gut microbiota varied considerably among individuals, there were nevertheless trends which could be identified across tuatara sampled for this study. The factor which explained the largest amount of variation in the microbiota was tuatara body condition, accounting for 23.9% of variation (*p* < .001). The sampling site explained 12.3% (*F* = 4.58, *p* < .001), its nested variable of maximum site temperature explained 7.3% (*p* < .01) and tick abundance explained 7.2% of variation (*p* < .01) (Figure 2). The PERMANOVA results reflected the importance of site to bacterial community composition and indicated that though life stage and sex explained less variation (2.5% and 4.3% respectively), these were also significant factors (*F*_life stage_ = 4.75, *p* < .001; *F*_sex_ = 2.69, *p* < .001) (Table 1).

**TABLE 1:** PERMANOVA for discrete variables (site, sex, life stage) and MANOVA for continuous variables (body condition, max. temperature, tick abundance) results showing factors which contributed significantly to tuatara gut microbiota composition.

	df	*F*	*R* ^2^	*p*
Site	5	4.58	.123	<.001
Sex	3	2.69	.043	<.001
Life stage	1	4.75	.025	<.001
Body condition			.239	.001
Max. temperature			.073	.003
Tick abundance			.072	.004

**FIGURE 3:**
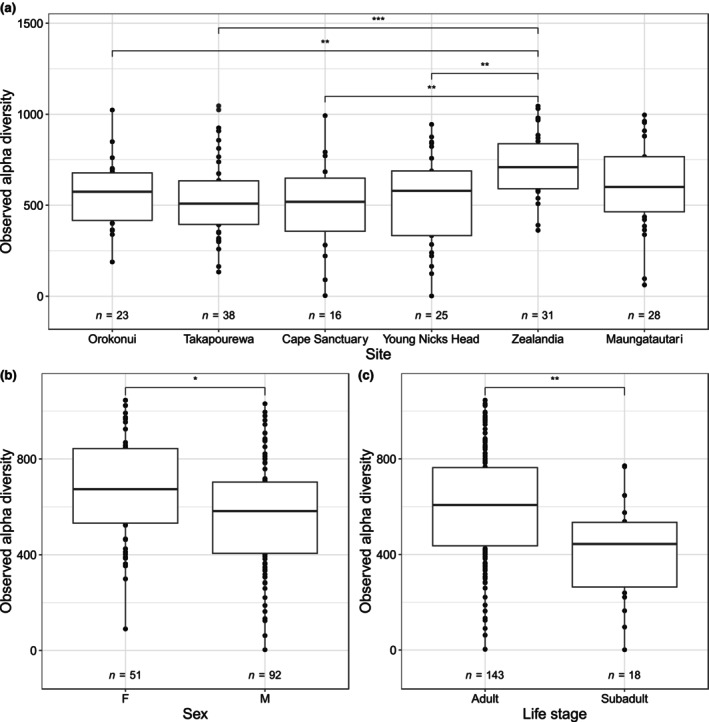
Observed bacterial alpha‐diversity in tuatara according to (a) sampling site, (b) sex and (c) life stage. *p < .05, **p < .01, ***p < .001, ****p < .0001.

Bacterial alpha‐diversity, measured by observed ASVs, differed significantly among some but not all sites. Tuatara at Zealandia Ecosanctuary exhibited significantly greater alpha‐diversity than those at Takapourewa, Orokonui Ecosanctuary, Young Nick's Head and Cape Sanctuary, but not Sanctuary Mountain Maungatautari. None of these sites aside from Zealandia Ecosanctuary varied significantly from one another, indicating a particularly high diversity of bacterial genera present at Zealandia Ecosanctuary (Figure 3a). Alpha‐diversity differed significantly between adult male and female tuatara (Figure 3b), and adult tuatara showed significantly greater alpha‐diversity than subadults (Figure 3c). It should be noted that the majority of tuatara sampled were adults, so this group had a much larger sample size than the subadults (143 adult tuatara compared with 18 subadults). Trends in alpha‐ and beta‐diversity were largely reflected when compared using Shannon alpha‐diversity (Figure S2) and weighted UniFrac to determine beta‐diversity (Figure S1, Table S1).

### 3.2 Taxonomy of the tuatara gut microbiota

**FIGURE 4:**
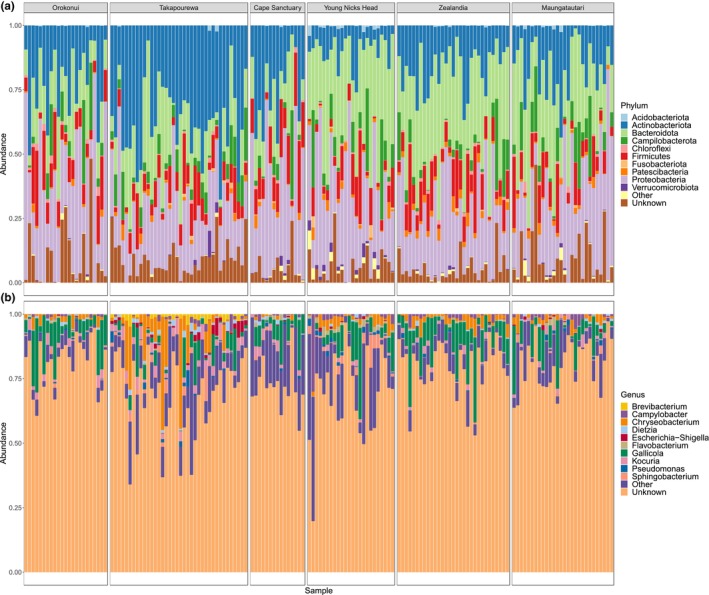
Dominant bacterial (a) phyla and (b) genera present in tuatara samples at each sampling site. ‘Other’ phyla are those represented in fewer than 20% of samples.

We sought to identify the bacterial taxa in the tuatara gut. The major phyla identified were *Proteobacteria* (30.7% of sequence reads across the entire data set), *Bacteroidota* (26.1%), *Actinobacteriota* (23.7%) and *Firmicutes* (10.1%), though the relative proportions of these varied within and among sites (Figure 4a). A majority of ASVs (72.8%) could not be assigned to genus level. Of those which were assigned, the majority were *Gallicola* (7.3% of total sequence reads), *Chryseobacterium* (3.3%), *Kocuria* (1.7%) and *Campylobacter* (1.0%) (Figure 4b). We identified 428 assigned bacterial genera, with 5000 unique ASVs unassigned at genus level.

With site significantly influencing the tuatara gut microbiota, we sought to determine which sites were more different from each other. Those sites with the lowest Jaccard similarity (indicating the most compositionally different microbiotas at ASV level) were Orokonui and Zealandia Ecosanctuaries (0.512), while the most similar sites were Zealandia and Cape Sanctuary (0.759) (Table 2).

**TABLE 2:** Bacterial community composition compared across sites using Jaccard similarities.

	Cape sanctuary	Sanctuary Mountain Maungatautari	Orokonui Ecosanctuary	Takapourewa	Young Nick's head
Sanctuary Mountain Maungatautari	0.6762				
Orokonui Ecosanctuary	0.6829	0.5220			
Takapourewa	0.7040	0.6029	0.62235		
Young Nick's Head	0.6875	0.6923	0.6871	0.7199	
Zealandia Ecosanctuary	0.7597	0.5679	0.5124	0.7085	0.7134

**FIGURE 5:**
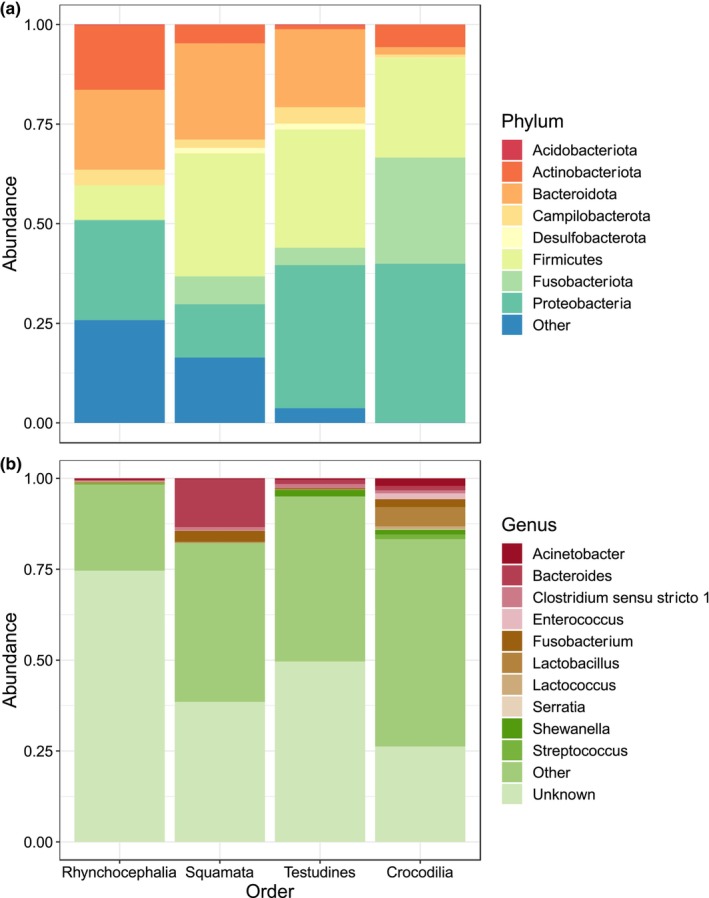
Dominant bacterial (a) phyla and (b) genera present in representatives of each reptile order which had publicly available microbiome data. Data for Squamata are derived from 61 host species; for Testudines 25 species; for Crocodilia 5 species.

When compared to the gut microbiotas of other reptiles, tuatara contained the lowest proportion of *Firmicutes*, the highest proportion of *Actinobacteriota* and the highest proportion of ASVs which could not be assigned to genus level (72%) among the four reptile orders (Figure 5). When compared using pairwise PERMANOVA, all orders contained significantly different bacterial communities, but rhynchocephalians were the most different from each of the other orders (Table 3).

**TABLE 3:** Pairwise PERMANOVA results for the comparison of each reptile order.

	Rhynchocephalia		Squamata		Testudines	
	*R* ^2^	*p*	*R* ^2^	*p*	*R* ^2^	*p*
Squamata	.169	<.001				
Testudines	.214	<.001	.007	<.001		
Crocodilia	.122	<.001	.004	.025	.008	.018

### 3.3 Identifying the presence of a core microbiota within tuatara

**FIGURE 6:**
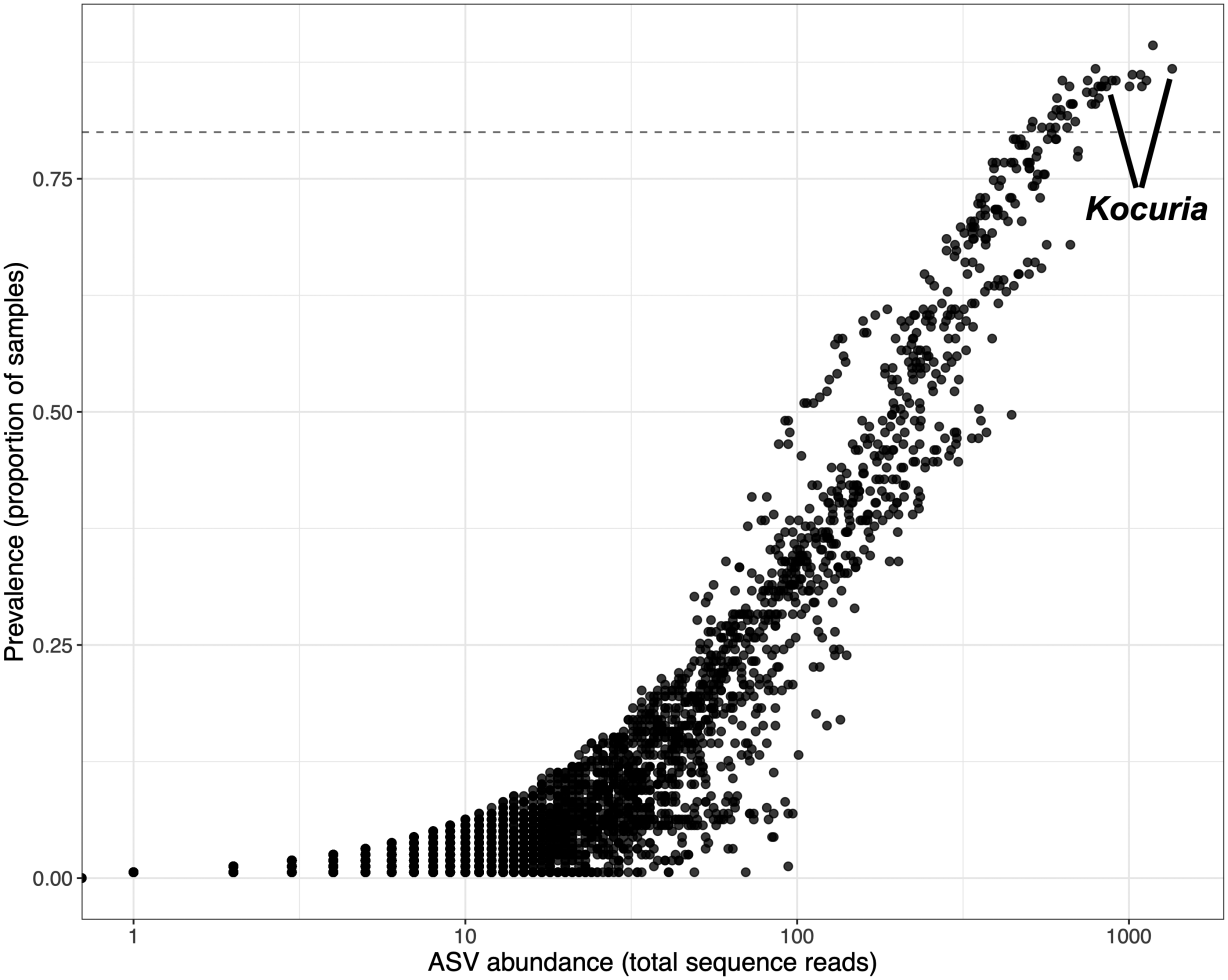
Prevalence and abundance of ASVs for tuatara across sites. The ‘core’ community was defined as those ASVs present in ≥80% of all tuatara sampled in this study. *Kocuria* is the only ASV in the core to be assigned at genus level, with the remaining 37 core ASVs all unassigned.

Using an 80% prevalence threshold, we identified 39 bacterial ASVs as members of the tuatara core microbiota across all studied sites. Of these 39, only two ASVs—both belonging to the genus *Kocuria*—could be assigned at genus level (Figure 6). When we examined site‐specific core microbiotas (i.e. those taxa present in at least 80% of samples from a given site), there were two ASVs in the core for Young Nick's Head, seven for Sanctuary Mountain Maungatautari, 32 for Cape Sanctuary, 80 for Takapourewa, 115 for Orokonui Ecosanctuary and 219 for Zealandia Ecosanctuary.

## 4 DISCUSSION

This study represents a first look at the bacterial community of the tuatara, the sole extant representative of the reptile order Rhynchocephalia. It is also among the largest microbiota data sets ever assembled for a reptile species, comprising the analysis of samples from 161 individual tuatara from six collection sites spanning a ~1000 km latitudinal gradient. Taken together, these data allowed us to explore key hypotheses surrounding (1) the gut microbiota of the rhynchocephalians compared with other reptiles, (2) the influence of biogeographic factors on the tuatara microbiota and (3) the extent to which factors such as host body condition, parasitism and ambient temperature affect the tuatara gut microbiota. We also identify an extensive core microbiota among tuatara, a strikingly high proportion of bacteria that could not be assigned at genus level, and reflect on the potential implications of our findings for tuatara conservation and management.

### 4.1 The tuatara gut microbiota is distinct from that of other reptiles

We hypothesised that, as rhynchocephalians, tuatara would have a distinct gut microbiota from the other three reptile orders. Our findings confirmed this, with tuatara demonstrating a higher level of unassigned genera than any other reptile order and significantly differing at the ASV level (Table 3). Of those relatively few bacterial genera within tuatara that could be assigned, we saw none of the most prevalent taxa from the other reptiles, with the genera *Gallicola* and *Kocuria* ranking most highly for tuatara but appearing only rarely if at all in the other reptile species (Figure 5). Interestingly, tuatara seemed to vary as much from the squamates as from any other order, suggesting the absence of a stronger link to their closest relatives.

Consistent with earlier, cultivation‐based work (Middleton et al., 2015), we failed to detect members of the bacterial genus *Salmonella* within tuatara, making tuatara distinct from other reptiles. Though they are readily exposed to *Salmonella* in the soil as well as from other animals sharing their burrows (Middleton et al., 2015), they appear to resist colonisation. As *Salmonella* is a well‐known community member in other reptile species (Jacobson, 2007; Lamm et al., 1972; Warwick et al., 2001), its apparent absence from tuatara suggests either a unique immune response on the part of the host or competitive exclusion by the resident microbiota. Elucidating the factors preventing *Salmonella* colonisation in tuatara is beyond the scope of this study, but warrants further attention as a trait separating tuatara from other reptile species and as a potential function for the tuatara gut microbiota.

### 4.2 Geographic location significantly influences the tuatara gut microbiota, but with many shared bacterial taxa across sites

As we hypothesised, the tuatara gut microbiota varied significantly across the six sampling sites, albeit to a lesser degree than expected, particularly considering the likely stochasticity associated with these low microbial biomass samples. Despite significant differences in bacterial communities among sites (Figure 2a), geographically disparate locations still shared as many as 75% of their respective ASVs (Table 2). As tuatara from each site have had no interaction with those from other sites since the initial translocation from Takapourewa more than a decade ago, we speculate that the translocated individuals have largely retained their gut microbiota in their new location and established a similar bacterial community in their offspring. An alternative interpretation is that tuatara have selectively recruited bacteria from their different environments to converge upon a similar microbial community profile.

The ecology of each sanctuary site ranged in temperature, humidity and distance from the coast. The presence of seabirds at a site was of particular interest, considering their ability to change soil chemistry and composition (Bellingham et al., 2010; Grant et al., 2022; Hawke, 2022). However, our study found no link between sites with or without seabirds (data not shown), and no link between sites which were closer geographically. As males on Takapourewa would also have finished a season where fairy prions made up a large part of their diet (Lamar et al., 2022), this indicates a lack of significant influence from expected sources such as diet, habitat and other species (Jin et al., 2021; Sullam et al., 2012; Zhang et al., 2021) with which the tuatara shares its burrow, further identifying tuatara as a species with unexpected drivers of its microbiota.

Within a site, individual tuatara primarily occupy a single burrow, which they inhabit during the day and leave at night to hunt and seek to reproduce during the summer/autumn mating season (Cree, 2014). There is currently little definitive information regarding the range of individual tuatara, though research has indicated that range is likely dependent on density and sex composition (Moore et al., 2009). Our data revealed little relationship between individual tuatara proximity and their bacterial community. Tuatara are more densely concentrated at some sampling sites than others, and our ability to sample across their full range at all sanctuaries was limited by accessibility. It is conceivable that tuatara which have more interactions (such as fighting, reproducing, or burrow swapping) share more bacteria. For example, the tuatara sampled at Zealandia Ecosanctuary were captured from within a relatively small area and were found to share more ‘core’ microbiota, though overall we did not find a clear linear relationship between sanctuary size and the size of the core microbiota. Orokonui Ecosanctuary contains ~300 hectares of tuatara habitat and we documented 115 core ASVs among the tuatara sampled there, whereas the much smaller Young Nick's Head sanctuary (~20 hectares) yielded a core microbiota comprising only 2 ASVs. Indeed, Young Nick's Head stands out as the least conserved site for microbiota, as tuatara here shared few of the ASVs found in the wider tuatara core (Figure 2a). While the interpretation of these data is limited by the area in which we were able to capture tuatara, it is compelling that the extent of a shared microbiota appears to be independent of tuatara density or proximity. The factors that may be driving the shared ‘core’ community of tuatara must include some element outside of physical interaction, such as diet, and indicate possibilities for how the tuatara obtain and retain their microbiota.

### 4.3 Tuatara body condition, parasitism and ambient temperature are all associated with composition of the tuatara gut microbiota

Tuatara body condition, calculated as the log ratio of snout‐vent length and weight, was predicted to be a significant driver of bacterial community composition. Microbiota differences during host disease or different body conditions have been established for a variety of animals, including humans and various reptiles (Duvallet et al., 2017; Filek et al., 2021), and our findings support this trend. Tuatara body condition was significantly associated with bacterial community composition, as was tick abundance (Figure 2b). Body condition is potentially linked to diet: tuatara eating a more nutritious diet may be consuming different dietary items and gaining better body condition as a result (Lamar et al., 2022), while also introducing different bacteria to their gut compared with tuatara of poorer body condition. Likewise, microbiota differences associated with parasitism could either indicate that parasitism changes the microbiota present in tuatara, or that tuatara with sub‐optimal microbiota are more vulnerable to parasitism. The microbiota is a known driver of immune response in some species (Zheng et al., 2020), and high levels of parasitism in tuatara could indicate a lack of some essential component of the gut microbiota.

Maximum temperature of the sampling site was also a significant driver of gut microbiota. We predicted that tuatara in higher temperatures may have an altered bacterial community, as in other reptile species (Moeller et al., 2020; Zhang et al., 2022). Indeed, ambient temperature did explain a significant amount of variation in the tuatara gut microbiota (Figure 2b). However, it is unclear whether there are specific microbes present or absent at sites with higher or lower temperatures (Figure 4) or if tuatara body temperature regulated by higher site temperatures allows for different microbes to persist. The coldest site, Orokonui Ecosanctuary, shared only 52% ASV similarity with the warmest, Sanctuary Mountain Maungatautari (Table 2), which indicates less similar microbiotas at the two sites at either end of the temperature spectrum. Further work will be required to identify specific functions of the bacteria present at either site, and to determine if there are potential health detriments for tuatara in warmer or colder locations based on their microbiota.

### 4.4 Future directions

In this study we analysed the V3–V4 region of the bacterial 16S rRNA gene using Illumina MiSeq, which typically provides sufficient taxonomic resolution for genus‐level assignment. However, for our tuatara data set only 28% of ASVs could be assigned to genus, with some (~12%) unassigned at even the phylum level. Future work employing long‐read sequencing technologies (e.g. Nanopore, PacBio) to obtain near full‐length 16S rRNA gene sequences could conceivably increase the rate of identification of these hitherto unassigned genera. Moreover, cultivation and shotgun metagenomics efforts should enable more insights into the likely functions of these potentially novel bacteria and their role in tuatara gut health.

All tuatara in our study were wild or in sanctuaries with minimal human interaction, though some sampling sites have more proximity to humans than others. Future sampling of tuatara in captivity, such as those in zoos, could provide more information about whether the ‘core’ microbiota we identified in this study persists in captive individuals and whether tuatara in captivity resemble other reptile hosts and experience a shift in their microbiota. Further study involving captive animals could also elucidate if tuatara on the relatively isolated Takapourewa have a different microbiota from tuatara which are handled rarely, like those in the sanctuaries, or from tuatara in captivity, which are handled more frequently. Studying animals in captivity also affords the opportunity to further explore the association between tuatara body condition and the gut microbiota, including establishing the directionality of this interaction. At present, it is impossible to tell from these data whether the microbiota significantly impacts host health or if host health shapes the gut microbiota.

## 5 CONCLUDING REMARKS

This study provides ample evidence that the microbiota of tuatara is as unusual and unique as the animal itself, and opens a number of doors for future study. There are a large number of currently unidentified bacteria in the tuatara gut, which serve unknown functions for the host reptile. We have also identified several drivers of tuatara gut microbiota composition, including geographic location and temperature, which have implications for where tuatara are translocated based on what temperatures are reached at these sites. These differences may not reflect significant repercussions for tuatara health, but may give us more information about how tuatara obtain, select and maintain a gut microbial community. This first look at the tuatara bacterial community gives us a glimpse into a microbiome potentially shared with ancient relatives, distinct from that of other reptiles and reiterates the status of tuatara as hosts of an unusual microbiome and immune system (Middleton et al., 2015; Waller et al., 2022). Tuatara are remarkable animals, which the indigenous Māori of Aotearoa New Zealand recognise as keepers of great knowledge and guardians of sacred places (Cree, 2014). Lamentably, they are also vulnerable to the effects of habitat loss, introduced predators and warming temperatures (Cree, 2014; Mitchell et al., 2008). This study is an important first exploration of the bacterial community of the tuatara, paving the way for future study of how tuatara interact with these bacteria, what their functions are, and how they are shaped by factors like diet and temperature.

## AUTHOR CONTRIBUTIONS

**Carmen Hoffbeck:** Conceptualization (equal); data curation (lead); formal analysis (lead); funding acquisition (lead); investigation (equal); methodology (equal); project administration (lead); visualization (lead); writing – original draft (lead); writing – review and editing (equal). **Danielle M. R. L. Middleton:** Conceptualization (equal); funding acquisition (equal); supervision (equal); writing – review and editing (equal). **Sarah K. Lamar:** Project administration (equal); writing – review and editing (equal). **Susan N. Keall:** Project administration (equal); writing – review and editing (equal). **Nicola J. Nelson:** Conceptualization (equal); investigation (equal); methodology (equal); project administration (equal); resources (equal); supervision (equal); writing – review and editing (equal). **Michael W. Taylor:** Supervision (equal); writing – original draft (equal); writing – review and editing (equal).

## FUNDING INFORMATION

University of Auckland Doctoral Scholarship to CH, James Fawcett New Zealand Herpetofaunal Postgraduate Research Award to CH. MBIE Strategic Science Investment Fund supported DMRLM.

## CONFLICT OF INTEREST STATEMENT

The authors declare that there are no competing interests in the publication of this work.

## Supporting information


Appendix S1

## Data Availability

The datasets presented in this study can be found in online repositories. The names of the repository/repositories and accession number(s) can be found at: https://www.ncbi.nlm.nih.gov/genbank/, PRJNA1008362. The workflow for analysis and visualisation in R is available in the supplement.
