# Supplementary material for: Gut microbiome of the sole surviving member of reptile order Rhynchocephalia reveals biogeographic variation, influence of host body condition and a substantial core microbiota in tuatara across New Zealand
Source: Ecol Evol. 2024 Feb 22;14(2):e11073. doi: 10.1002/ece3.11073 (PMC10884523; doi:10.1002/ece3.11073)
Supplement: Supplementary file 1 — Appendix S1 [file ECE3-14-e11073-s001.docx]

**Supplemental Material for:**

**Gut microbiome of the sole surviving member of reptile order Rhynchocephalia reveals biogeographic variation, influence of host body condition, and a substantial core microbiota in tuatara across New Zealand**

Carmen Hoffbeck^1^, Danielle M.R.L. Middleton^2^, Sarah K. Lamar^3^, Susan N. Keall^3^, Nicola J. Nelson^3^, Michael W. Taylor^1,^*

^1^ School of Biological Sciences, University of Auckland, Auckland, New Zealand

^2^ Manaaki Whenua – Landcare Research, Lincoln, New Zealand

^3^ School of Biological Sciences, Victoria University of Wellington, Wellington, New Zealand

* Corresponding author: mw.taylor@auckland.ac.nz


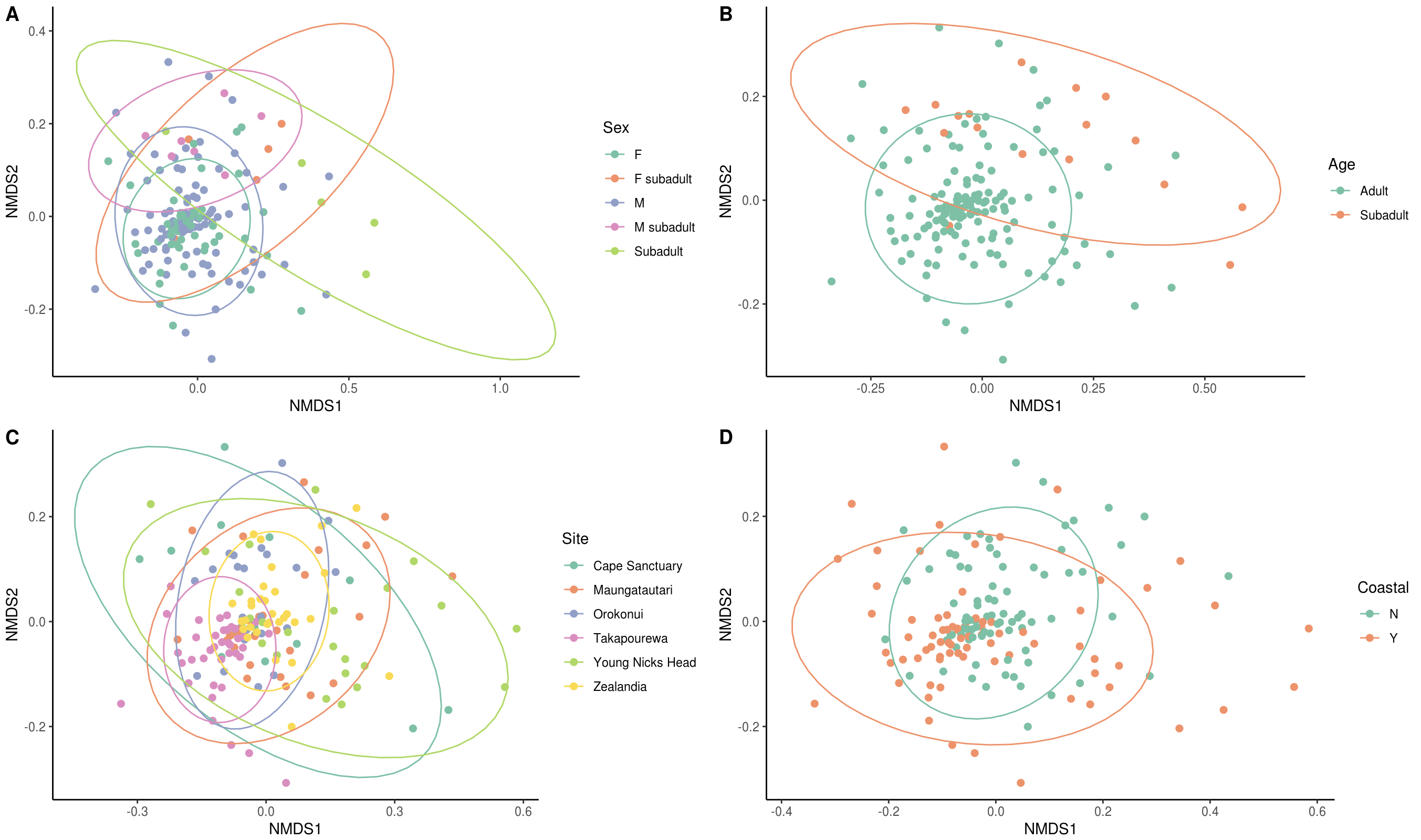


Figure S1. 16S rRNA gene-based nMDS ordination showing effects on tuatara bacterial community composition of (A) sampling site, and (B) tuatara body condition, tick abundance, and maximum temperature at the sampling site using weighted UniFrac. Ellipses in (A) represent statistically similar communities at 95% confidence. Arrow length in (B) represents the strength of the R^2^ value for that factor.

Table S1. PERMANOVA for discrete variables (site, sex, life stage) and MANOVA for continuous variables (body condition, max. temperature, tick abundance) results showing factors which contributed significantly to tuatara gut microbiota composition using weighted UniFrac.

|  | df | F | R^2^ | p |
| --- | --- | --- | --- | --- |
| Site | 5 | 7.60 | 0.184 | < 0.001 |
| Sex | 3 | 3.60 | 0.034 | < 0.001 |
| Life stage | 1 | 7.03 | 0.033 | < 0.001 |
| Body condition |  |  | 0.059 | 0.012 |
| Max. temperature |  |  | 0.070 | 0.005 |
| Tick abundance |  |  | 0.033 | 0.058 |

Figure S2. Bacterial alpha-diversity using the Shannon metric in tuatara according to (A) sampling site, (B) sex, and (C) life stage. * p < 0.05, ** p < 0.01, *** p < 0.001, **** p < 0.0001.
